# Characterization and Laser Structuring of Aqueous Processed Li(Ni_0.6_Mn_0.2_Co_0.2_)O_2_ Thick-Film Cathodes for Lithium-Ion Batteries

**DOI:** 10.3390/nano11071840

**Published:** 2021-07-16

**Authors:** Penghui Zhu, Jiahao Han, Wilhelm Pfleging

**Affiliations:** Institute for Applied Materials-Applied Materials Physics (IAM-AWP), Karlsruhe Institute of Technology (KIT), Hermann-von-Helmholtz-Platz 1, 76344 Eggenstein-Leopoldshafen, Germany; jiahao.han@student.kit.edu

**Keywords:** lithium-ion batteries, NMC 622, aqueous processing, thick-film electrode, laser structuring, cyclic voltammetry, rate capability

## Abstract

Lithium-ion batteries have led the revolution in portable electronic devices and electrical vehicles due to their high gravimetric energy density. In particular, layered cathode material Li(Ni_0.6_Mn_0.2_Co_0.2_)O_2_ (NMC 622) can deliver high specific capacities of about 180 mAh/g. However, traditional cathode manufacturing involves high processing costs and environmental issues due to the use of organic binder polyvinylidenfluoride (PVDF) and highly toxic solvent *N*-methyl-pyrrolidone (NMP). In order to overcome these drawbacks, aqueous processing of thick-film NMC 622 cathodes was studied using carboxymethyl cellulose and fluorine acrylic hybrid latex as binders. Acetic acid was added during the mixing process to obtain slurries with pH values varying from 7.4 to 12.1. The electrode films could be produced with high homogeneity using slurries with pH values smaller than 10. Cyclic voltammetry measurements showed that the addition of acetic acid did not affect the redox reaction of active material during charging and discharging. Rate capability tests revealed that the specific capacities with higher slurry pH values were increased at C-rates above C/5. Cells with laser structured thick-film electrodes showed an increase in capacity by 40 mAh/g in comparison to cells with unstructured electrodes.

## 1. Introduction

Lithium-ion batteries (LIBs) have been widely applied in energy storage applications, especially in personal portable electronic devices, due to their high energy density, long cyclic stability, and good safety [1,2,3,4,5]. Despite the huge success and continuous growing specific energy at 6%/year [6], there are still several technical challenges for the application of LIBs in automotive industries in order to meet or exceed the requirements compared to conventional vehicles with internal combustion engines, such as long cycle life (>500 cycles), a calendar life >10 years, and stability at different temperatures ranging from −30 to 52 °C [7]. The production costs of LIBs, currently about 250 USD/kWh, still limit their widespread use in the automobile industry and should be reduced to below 125 USD/kWh [7,8].

There are two possible approaches for a further significant reduction of the battery costs. The first method is to lower the electrode processing costs, which are associated with the expensive organic solvent and the drying process of the wet electrode. Polyvinylidenfluoride (PVDF) is a state-of-the-art binder in commercial LIBs, especially for cathodes. It must be dissolved by a volatile solvent named *N*-methylpyrrolidone (NMP), which is not only expensive but also highly toxic. Therefore, extra ventilation and filtration devices are necessary for the protection of production lines and workers’ safety [9]. In comparison to organic binders, aqueous binders are environmentally benign and have about one order of magnitude lower costs than PVDF [10]. During aqueous processing, water serves as a solvent for the preparation of electrode slurry, which lowers the material costs and is also favorable for the environment. The removal of water during drying is 4.5 times faster than for NMP and requires nearly 10 times less overall energy for the processing [11].

Water-based binders such as sodium carboxymethyl cellulose (Na-CMC) and styrene butadiene rubber (SBR) have been successfully established for the production of graphite and silicon/graphite anodes [12,13,14]. Recently, numerous researchers have focused on the development of the aqueous processing of cathode materials. Chen et al. [15] reported the excellent high rate capability and stable cycling performance of Li(Ni_0.4_Mn_0.4_Co_0.2_)O_2_ (NMC 442) using Na-CMC as binder compared to electrodes with PVDF binder. Çetinel and Bauer et al. [16,17] studied the rheological properties and electrochemical performance of Li(Ni_1/3_Mn_1/3_Co_1/3_)O_2_ (NMC 111) with Na-CMC and fluorine acrylic hybrid latex (TRD 202A) with different amounts of carbon black and binder, while Loeffler et al. [18] characterized pouch cells consisting of aqueous processed NMC 111 cathode and graphite anode with 70% capacity retention after 2000 cycles. Other cathode materials such as LiCoO_2_ (LCO), LiFePO_4_ (LFP), LiNi_0.80_Co_0.15_Al_0.05_O_2_ (NCA), Li(Ni_0.5_Mn_0.3_Co_0.2_)O_2_ (NMC 532) have been reported using aqueous binders [19,20,21,22,23]. In addition, guar gum and poly acrylic acid (PAA) binders were applied for cathode manufacturing [17,24]. However, lithium and transition metal leaching from the active material into water and current collector corrosion due to increased slurry pH values are the major challenges for the aqueous processing of cathodes [21].

Another way to lower the production costs of LIBs is to increase the electrode film thickness. The state-of-the-art cathodes are limited to 100 µm thickness with a mass loading of about 20 mg/cm^2^ after being deposited onto the current collector [25]. A higher film electrode thickness, i.e., a higher areal capacity, is preferred since this not only increases the electrode specific capacity but also reduces the proportion of inactive materials in batteries. The costs of other manufacturing steps during assembling such as cutting, welding, and stacking, will be reduced [9]. However, the lithium-ion diffusion kinetics inside thick-film electrodes will be the bottleneck to enable high power operation at high charging/discharging rates [26]. In order to solve this problem, structuring of thick-film electrodes (“3-dimensional battery”) using ultrafast laser ablation opens a new path to simultaneously increase energy density and power density [27,28,29,30].

Many researchers have studied the effect of different types of acid addition on the cathode materials [31,32,33,34]. However, only a few groups have studied the effect of pH value on the electrochemical properties of electrodes. Ibing et al. [35] studied the effect of pH values at 12.5 and 7.6 on the electrochemical performance of NMC 532 electrodes, while Bauer et al. [17] reported the performance of aqueous processed NMC 111 electrodes at pH values of 7–11. So far to the best of our knowledge, no related research was established for NMC 622. Therefore, in the present work, NMC 622 cathodes were manufactured using aqueous binders CMC and TRD 202A. Different amounts of acetic acid were applied during the mixing process in order to investigate the effect of slurry pH value on the electrochemical performance of electrodes assembled in half cells. In addition, ultrafast laser structuring of thick-film electrodes was applied to prove the feasibility of the 3D battery concept for aqueous processed electrodes.

## 2. Materials and Methods

### 2.1. Electrode Preparation

Commercially available NMC 622 powder (BASF SE, Ludwigshafen, Germany) with a particle size of 12.8 µm (D90) was used as an active material for cathode, while carbon black C-NERGY Super C65 (Imerys G & C Belgium, Willebroek, Belgium) was used as a conductive additive. Na-CMC (CRT 2000PA, Doe Wolff Cellulosic, Bomlitz, Germany) with a substitution degree (the average number of substituent groups attached per base unit) of 0.82–0.95 and water-based fluorine acrylic copolymer latex TRD 202A (JSR Micro NV, Leuven, Belgium) were applied as binders for aqueous electrode preparation. The electrode slurries were mixed in a dissolver equipped with a vacuum pump (CDS, VMA-Getzmann, Reichshof, Germany). NMC 622, carbon black, and deionized water were added into a premixed 5 wt.% CMC solution and were mixed under vacuum using 2000 RPM rotation speed for 20 min. After that, acetic acid was immediately added to the slurry in order to adjust the pH value. The slurry was further stirred for 5 min under vacuum using the same rotation speed as in the previous step, followed by 90 min mixing without vacuum. Finally, TRD 202A was added to the slurry and stirred with 500 rpm under vacuum for 3 min, since TRD 202A is shear sensitive. The solid components were adjusted to a mass ratio of NMC 622:carbon black:Na-CMC:TRD 202A = 100:2:2:3. The pH value of the slurry was measured 10 min after the mixing process was finished via a pH-Meter FE30-Basic FiveEasy (Mettler-Toledo GmbH, Giessen, Germany). The viscosity of the slurries was measured at room temperature with a rheometer (MCR 72, Anton Paar GmbH, Graz, Austria) using the rotational flow measurements with a parallel plate (PP 50, Anton Paar GmbH, Graz, Austria).

The slurry obtained from previous steps was tape cast with a doctor blade (ZUA 2000.100, Proceq, Schwerzenbach, Switzerland) onto a 20 µm aluminum-foil using a tape casting coater (MSK-AFA-L800-LD, MTI Corporation, Richmond, CA, USA) with vacuum pump. The coating speed was set at 5 mm/s and the thickness of the coated cathode film was controlled by varying the gap of the doctor blade. The cast electrodes were dried at room temperature to avoid possible accelerated chemical corrosion which might occur at elevated temperatures. The thicknesses of dried cathodes were kept at 186–240 µm (mass loading: 34–47 mg/cm^2^) for thick-film and 50–130 µm (mass loading: 10–14 mg/cm^2^) for thin-film electrodes, respectively. Afterward, the electrodes were calendered using an electric precision rolling press (MSK-2150, MTI Corporation, Richmond, CA, USA) at room temperature with a constant calendering speed of 35 mm/s. The average porosity of the thick-film NMC 622 electrodes was adjusted to 35%, while the thicknesses of thin-film cathodes were reduced by 10–15%. The porosity of the electrodes was calculated based on the weight and density of each component [36].

Afterward, ultrafast femtosecond (fs) fiber laser (Tangerine, Amplitude Systèmes, Pessac, France) with a pulse duration of 380 fs and an operational wavelength of 515 nm (M^2^ < 1.2) was applied to structure selected thick-film electrodes. Line structures with 200 µm pitch and with depths down to the current collector were generated. A repetition rate of 500 kHz and an average laser power of 4.27 W were applied, while the laser scanning speed was kept constant at 500 mm/s and the number of scan passes was varied from 30 to 50 with regard to the applied cathode film thickness. Cross-sectional analyses of electrodes using different scan passes were performed in order to find appropriate structuring parameters. Samples with a size of 2 cm × 1.3 cm (width × height) were cut using an ultrafast laser from the calendered cathode sheets. Then the samples were held vertically with a plastic clip and were placed in a holder and were afterward filled with resin, hardener, as well as fluorescent powder, to acquire metallographic samples. Afterward, the samples were ground and polished. The electrodes were cut in circles with 12 mm in diameter for coin cell design using 200 mm/s scanning speed and 10 scan passes, while the other parameters remained unchanged with regard to laser structuring. The laser processing was performed in ambient air.

### 2.2. Cell Assembly and Electrochemical Analysis

NMC 622 cathodes were assembled versus 0.25 mm lithium foil (Merck KGaA, Darmstadt, Germany) in coin cells CR2032 in an argon-filled glove box (LAB master pro sp, M. Braun Intergas-Systeme GmbH, Garching, Germany) with H_2_O < 0.1 ppm and O_2_ < 0.1 ppm. A mixture of ethylene carbonate and ethyl methyl carbonate (EC/EMC 3:7) with 1.3 M lithium hexafluorophosphate (LiPF_6_) as conducting salt and 5 wt.% fluoroethylene carbonate (FEC) as additive was used as electrolyte. The separator was polypropylene (PP, Celgard, Charlotte, NC, USA) foil with a thickness of 25 µm and a diameter of 15 mm. A total amount of 120 µL electrolyte was added to the cell. After stacking, all cell components were pressed using a digital pressure controlled electric crimper (MSK-160D, MTI Corporation, Richmond, CA, USA). The assembled batteries were stored at room temperature for 20 h in order to allow the cathode and separator to be fully wetted with liquid electrolyte.

The “constant current–constant voltage” (CCCV) method was carried out for rate capability tests using a battery cycler (BT 2000, Arbin Instruments, College Station, TX, USA). The first 3 cycles were carried out at C/20 as the formation step, followed by increasing C-rates from C/10 to 5C. C/10 and C/5 were carried out for 5 cycles and the others were carried out for 10 cycles. The C-rate was calculated based on the discharge time and applied current during the formation step. In this work, 1C = 0.99 h^−1^, as well as a specific capacity of 155 mAh/g, were applied for the calculation of currents under different C-rates. The lower and upper cut-off voltages were 3.0 and 4.2 V, respectively. The CV measurements with a scan rate at 0.02 mV/s were carried out to find out the redox reaction that occurred during the charge and discharge processes. The operating window was set to 3.0 to 4.3 V. The electrochemical analyses were performed at room temperature in an ambient atmosphere.

## 3. Results

### 3.1. Characterization of Slurries and Electrodes

**Figure 1:**
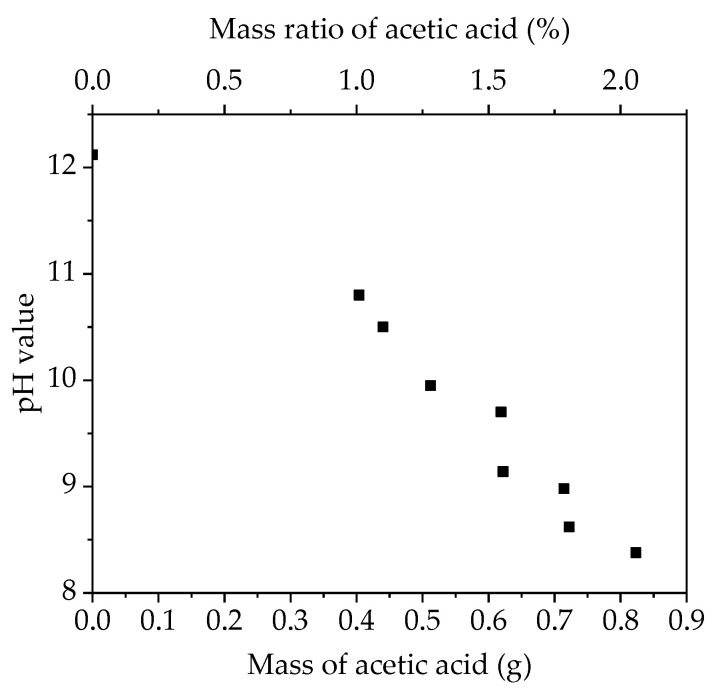
The pH value of slurry as a function of the mass and mass ratio of added acetic acid.

In order to adjust the pH value, acetic acid was added to the slurry. Since the pH value of slurries with an aqueous binder increased with time [21], the slurry pH values were measured 10 min after slurry completion. As shown in Figure 1, the pH value of the slurry linearly decreases with the amount of added acetic acid. The mass ratio of acetic acid given in Figure 1 is referred to as the mass ratio of acetic acid and NMC 622.

**Figure 2:**
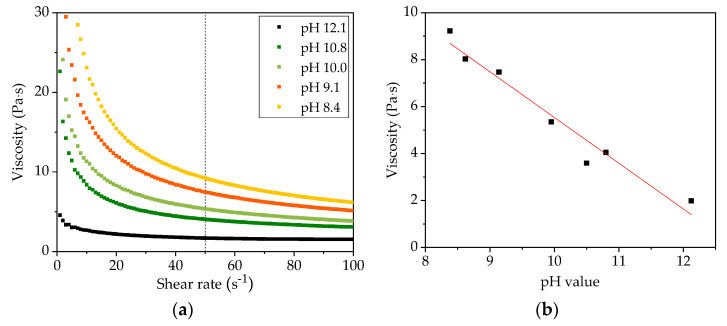
The viscosity of slurries as a function of (**a**) shear rate and (**b**) pH value.

Before tape casting, the viscosity of slurries with different pH values was measured at shear rates from 0 to 100 s^−1^. As shown in Figure 2a, slurries with different pH values show similar shear thinning behavior which means that the viscosity decreases with increasing shear rate. This indicates the existence of soft agglomerates within the slurries. With decreasing slurry pH value, a distinct increase of viscosity was found, suggesting that higher shear force is needed to break the agglomerates in slurries with lower pH values. Figure 2b shows the viscosity of slurries with different pH values at a reference shear rate of 50 s^−1^. The functional relationship between viscosity and pH value shows approximately linear correlation. During experiments, it was found that slurries with a viscosity of 3 to 8 Pa·s at 50 s^−1^ were beneficial to prepare thick-film electrodes. In general, the manufacturing of thick-film electrodes can be achieved by reducing the solvent (water) content or by increasing the binder content in slurries, which in both cases leads to an increase in viscosity.

Slurries with different pH values were tape cast on 20 µm Al-foil and dried in ambient air. The details of the electrodes are summarized in Table 1. Thick-film electrodes with mass loading from 37 to 46 mg/cm^2^ were manufactured, while thin-film electrodes with about 11 mg/cm^2^ (1/4 in comparison to thick-film electrodes) were used to represent electrodes with a state-of-the-art film thickness. It was found that without acid addition, the cathodes have a high porosity of about 60%. For example, the cathode with a slurry pH value of 12.1 shows a 50 µm thicker layer in comparison to the cathode with a slurry pH value of 10.8, while the mass loading is reduced by 6 mg/cm^2^.

**Figure 3:**
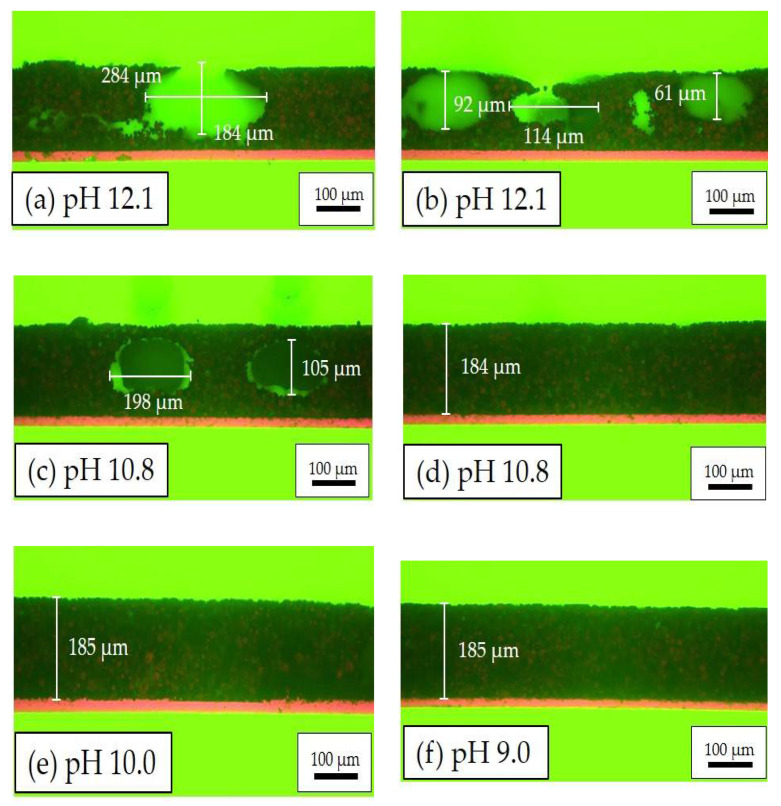
Microscope images of electrodes in cross-sectional view with different slurry pH values: (**a**,**b**) pH = 12.1; (**c**,**d**) pH = 10.8; (**e**) pH = 10.0; and (**f**) pH = 9.0.

Cross-sectional analyses were performed with electrodes whose slurries had different pH values, see Figure 3. In the electrode film with a slurry pH value of 12.1, many cavities with diameters ranging from 60 to 284 µm were formed owing to the generation of H_2_, which is produced when aluminum is directly exposed to the alkaline slurry [19]. The cavity structures are distributed across the electrode film, leaving openings with diameters from 25 to 105 µm on the electrode surface. When the slurry pH value decreases to 10.8, the diameters of cavities are reduced to 104 to 198 µm, as shown in Figure 3c. Meanwhile, the amount of cavities is decreased. For example, Figure 3d shows a segment of the cathode with a slurry pH value of 10.8 without obvious cavities. With further decreasing of slurry pH values to 10.0 and 9.1, the electrodes become flat without any cavities, as shown in Figure 3e,f.

**Figure 4:**
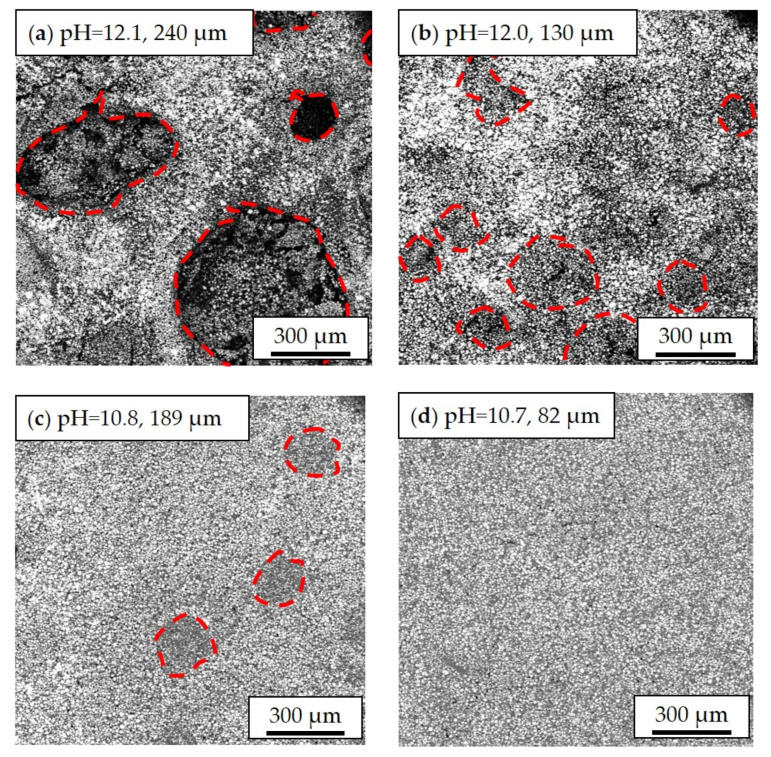
Scanning electron microscope (SEM) images of cathode films with different slurry pH values and different thicknesses: (**a**) pH = 12.1, 240 µm; (**b**) pH = 12.0, 130 µm; (**c**) pH = 10.8, 189 µm; (**d**) pH = 10.7, 82 µm. The craters on the electrode surface with different diameters are marked with red dot lines.

In addition to the slurry pH values, the film thickness is another decisive factor that influences electrode homogeneity. Inhomogeneous distribution of cathode materials is observed in thick-film electrodes (Figure 4). The dark regions marked with red circles are craters that were created by the inward collapse of electrode materials during the drying process. For thick-film cathodes (slurry pH value 12), craters with diameters ranging from 150 µm to 700 µm have been formed. By decreasing the slurry pH value to 10.8, the craters on the surface became smaller and shallower with diameters of 190 to 200 µm. As for thin-film electrodes with a slurry pH value of 12.0 and a thickness of 67 µm, the crater diameters were around 80 to 150 µm which is about a quarter in size compared to those in thick-film electrodes. In thin-film electrodes with slurry pH values of 10.7, the active material particles were uniformly distributed. No obvious cavities were found on the film surface. This proves that the corrosion of the current collector in thick-film electrodes has more impact in comparison to thin-film electrodes which is due to the longer drying time of thick-film electrodes.

For the purpose of improving lithium-ion diffusion kinetics, thick-film electrodes with slurry pH values of 12.1, 10.0, and 9.0 were structured via ultrafast laser ablation prior to cell assembling and subsequent galvanostatic measurements. The data of laser structured electrodes are summarized in Table 2. The mass loss due to laser ablation includes the removal of active material, binder, and conductive additive.

**Figure 5:**
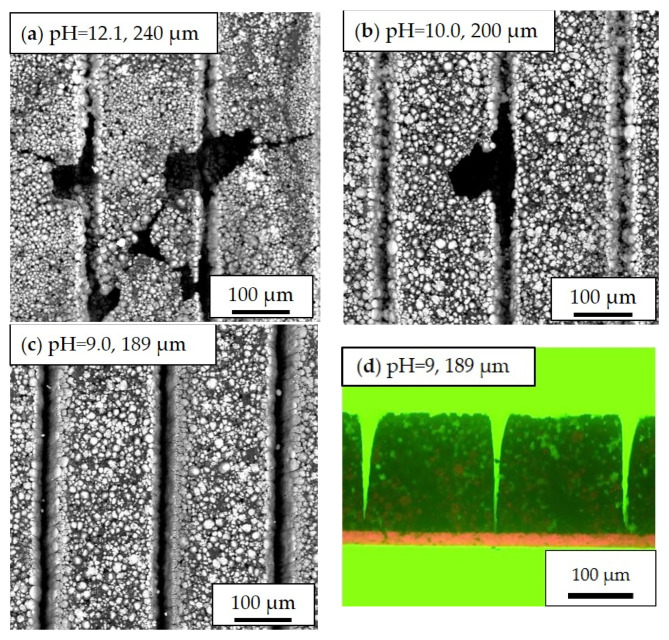
SEM images of structured cathodes with slurry pH values of (**a**) 12.1; (**b**) 10.9; and (**c**) 9.0; (**d**) Microscope image of cross sectional view of a laser structured cathode deposited with a slurry pH value of 9 and a film thickness of 189 µm.

The SEM images of laser structured electrodes (slurry pH values of 12.1, 10.0, and 9.0) are shown in Figure 5. On the surface of electrodes manufactured with a slurry pH value of 12.1, holes with diameters varying from 10 µm to 100 µm were observed. Furthermore, cracks originated from the holes that were formed. As the slurry pH value decreased to 10.0, no crack formation and holes with diameters larger than 50 µm were obtained. At a slurry pH value of 9.0, no holes or craters were found in the entire electrode. Figure 5d shows the line structure with 200 µm pitch which was generated using ultrafast laser ablation without damaging the aluminum current collector. A width of 30 ± 2 µm is achieved at the top of the groove, and the half-peak-width is 10 ± 1 µm. The sidewalls of the generated grooves show a slight curvature of 7.0° ± 0.3° and the orientation is perpendicular to the cathode surface. The laser fluence calculated from pulse energy and laser focus diameter is 14.7 J/cm^2^, while the pulse overlap is 96.7%. The active mass loss owing to the laser structuring process amounts 3% to 10%. No obvious debris formation is found on the electrode surface and inside the channel structures.

### 3.2. Electrochemical Performance of Cells

Thick-film and thin-film NMC 622 electrodes with different slurry pH values were assembled vs. lithium in coin cells. Laser structured thick-film electrodes with pH 12.1, 10, and 9 were assembled in order to study the effect of laser structuring on cell performance. The results from the rate capability test as well as cyclic voltammetry are presented and discussed.

#### 3.2.1. Rate Capability Test

**Figure 6:**
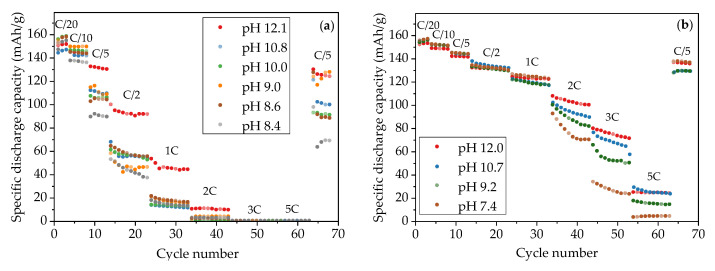
Specific discharge capacity of cells containing (**a**) thick-film electrodes and (**b**) thin-film electrodes produced with different slurry pH values.

The specific discharge capacity of water-based NMC 622 electrodes is shown in Figure 6. In the formation step at C/20, the specific capacities of all cells with thick-film electrodes are slightly increased. For example, for cells containing electrodes produced with slurry pH values of 10.0, 9.0, and 8.6, the specific capacity increased from 153 mAh/g to 158 mAh/g after formation, while cells containing cathodes produced with slurry pH values of 12.1 and 10.8 achieve about 150 mAh/g capacity at the third C/20 cycle. At C/10, the cell with the electrode produced with a slurry pH value of 9.0 retains the highest specific capacity of 150 mAh/g, while the cell with the electrode produced with a slurry pH value of 8.4 shows the lowest specific capacity of 136 mAh/g. As the C-rate rises to C/5 and C/2, cells containing electrodes produced with a slurry pH value of 12.1 hold the highest capacity of 130 mAh/g and 91 mAh/g at C/5 and C/2, respectively, while the lowest specific capacities (90 mAh/g at C/5 and 37 mAh/g at C/2) belong to the electrode produced with a slurry pH value of 8.4. At 1C and 2C, all cells with thick-film electrodes show a drop in capacity by more than 80% with regard to the initial capacity, except those cells containing electrodes that were produced without acid addition (slurry pH value 12.1). The cell with the electrode produced without acid addition maintains the highest capacity of 35 mAh/h at 1C and 9 mAh/g at 2C. For discharge rates of 3C and 5C, all cells with thick-film electrodes no longer show any capacity.

In order to verify the capacity retention of electrodes after being cycled at high discharge rates up to 3C, the C-rate was reduced to C/5. All cells show less specific capacity compared to the previous C/5 cycles. The cell with the electrode produced with a slurry pH value of 12.1 provides the highest specific capacity of 124 mAh/g with a capacity retention of 95%, while the lowest capacity belongs to the electrode produced with a slurry pH value of 8.4 with a specific capacity of 69 mAh/g and 77% capacity retention.

The specific capacities of cells with thin-film electrodes are higher than cells containing thick-film electrodes produced with the same slurry pH value (Figure 6b), especially at high discharge rates (≥C/2). After the C/20 formation step, all cells achieve similar specific capacities of 153–157 mAh/g, which are similar to those obtained with thick-film electrodes. With increasing C-rate, the specific capacity of all cells shows successive deterioration. For C/10 up to 1C, cells with thin-film electrodes show similar discharge capacities: 149–152 mAh/g at C/10, 143 mAh/g at C/5, 133 mAh/g at C/2, and 120 to 125 mAh/g at 1C. In this C-rate range, electrodes produced with different slurry pH values show no significant difference. However, from 2C to 5C, the capacity of thin-film electrodes decreases with increasing cycle number, especially for electrodes that were produced with a slurry pH value of 7.4. The drop in capacity at high C-rates with decreasing slurry pH values is also observed in cells with thick-film electrodes. For the final C/5 cycles, the electrodes produced with slurry pH values of 12.0 and 7.4 show the highest reversible specific capacity of 137 mAh/g, which had 95% capacity retention compared to the initial C/5 cycles (144 mAh/g), while other cells containing cathodes produced with slurry pH values of 10.7 and 9.2 show lower reversible specific capacity of 130 mAh/g with capacity retention of 90%.

**Figure 7:**
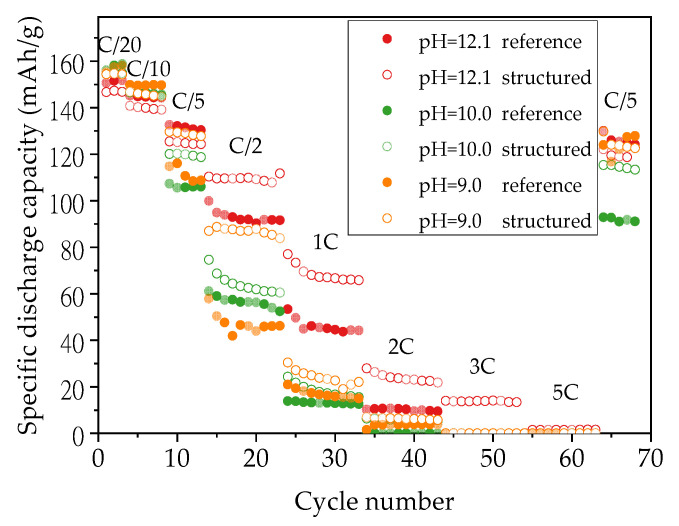
Specific discharge capacity of cells containing unstructured and laser structured thick-film electrodes which were produced with different slurry pH values.

Figure 7 shows the discharge capacities of cells containing structured thick-film electrodes which were produced with slurry pH values of 12.1, 10.0, and 9.0. During the formation step and C/10 cycles, all cells with structured electrodes show discharge capacities reduced by 4 mAh/g in comparison to cells with unstructured electrodes produced with the same slurry pH values and same film thicknesses. In following cycling steps at C/5, a capacity increase of 14 mAh/g and 18 mAh/g is observed for cells containing structured cathodes produced with slurry pH values of 9.0 and 10.0 in comparison to cells with unstructured electrodes, respectively, while cells with laser structured electrodes without acid addition have 7 mAh/g lower capacity than cells with unstructured electrodes. However, when the C-rate is increased above C/2, laser structured electrodes begin to show a great improvement regarding specific discharge capacity retention regardless of the slurry pH values used during electrode preparation. The cell with structured electrode produced with a slurry pH value of 12.1 retains the highest discharge capacity from C/2 to 3C, while the cell containing structured cathode produced with a slurry pH value of 10.0 shows 46% higher capacity at C/2 compared to the cell with an unstructured cathode. After being cycled at elevated C-rates, the cell with the structured cathode (slurry pH value of 12.1) shows a capacity retention of 97% compared to the previous first cycle at C/5, while cells produced with slurry pH values of 10.0 and 9.0 maintain 96% discharge capacity.

#### 3.2.2. Cyclic Voltammetry

**Figure 8:**
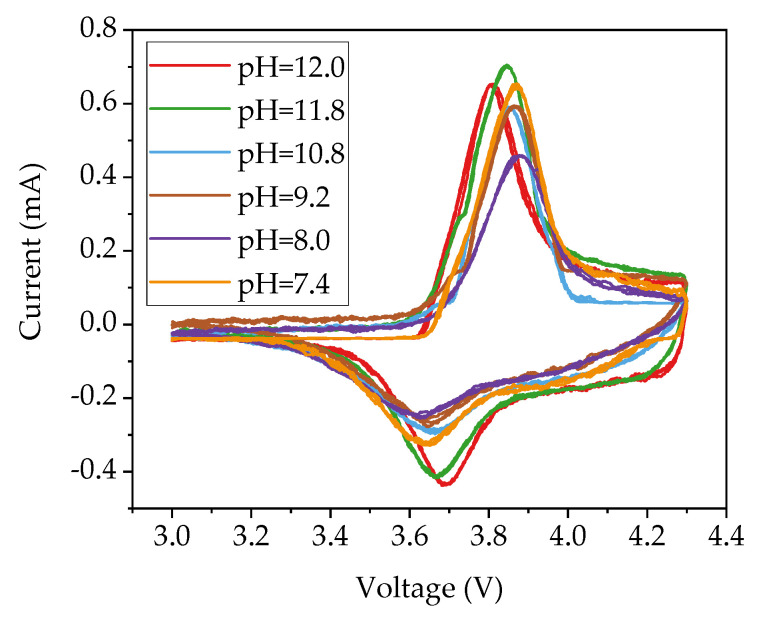
CV plots of cells containing cathodes that were produced with different slurry pH values.

CV measurements of cells with unstructured cathodes were performed in order to investigate the impact of the added amount of acetic acid on the electrochemical reaction of the active material. The current peaks in CV correspond to redox reactions and can be assigned to different phase transitions. The recorded signals were fitted using the Gaussian window function in MATLAB (MathWorks Inc., Version: R2019b, Natick, MA, USA). The recorded current signals in Figure 8 during the charging and discharging process formed a closed loop with hysteresis. For each sample, the measured three cycles for each cell overlap with each other, which proves quite good cycle stability of the prepared electrodes.

During each charging and discharging process, only one current peak is clearly observed, which indicates on the cathode side the presence of an oxidation and reduction reaction, respectively. In the charging process, the peak maximum shifts from 3.80 V to 3.86 V with decreasing slurry pH value from 12.0 to 10.8. However, when the slurry pH value decreases further below 10.8, the corresponding peak voltage remains at 3.87 V. In discharging process, the maximum peak shifts from 3.70 V to 3.66 V voltage with decreasing slurry pH value to 10.8, while the voltage of the peak maximum remains unchanged at 3.64 V for cells with electrodes produced with slurry pH values from 9.2 to 7.4.

## 4. Discussion

The effect of slurry pH value on electrode processing as well as on the electrochemical performance of cells with dried electrodes is discussed in detail. Cells with laser structured electrodes show higher specific capacity in comparison to cells with unstructured electrodes. This confirms the conducive effect of the 3D battery concept with the combination of a thick-film electrode and laser structuring.

### 4.1. Effect of Acid Addition on The Electrode Processing

During aqueous processing, the pH value of NMC 622 slurries without acid addition reached 12.1 after the mixing process, which is similar to already published results [17,37]. The increase of the slurry pH value is due to the Li^+^/H^+^ exchange mechanism between layered oxide electrode materials and water, which leads to the formation of lithium hydroxide (LiOH) and other products such as LiHCO_3_ and Li_2_CO_3_ [38]. Since the cation exchange only takes place on the edges of the active material, a part of the protons in the surface of NMC 622 can be swapped with Li^+^ upon cycling. This may explain the increase of the discharge capacity of all cells with aqueous-based cathodes during the formation step, as shown in Figure 6. When exposed to alkaline slurries, a local breakdown of the natural oxide layer on the aluminum current collector takes place, leading to the corrosion of the aluminum substrate and hydrogen formation [39,40,41]. The generation of the hydrogen along the interface between the current collector and slurry results in a high porosity with values above 57% and cavities having a diameter larger than 100 µm inside the electrodes after the drying process, as shown in Figure 3a. The size and number of pores inside the electrode are assumed to depend not only on the slurry pH value but also on wet film thickness (doctor blade height) and drying temperature. In this work, all electrodes were dried at room temperature. Therefore, the influence of temperature can be neglected. With increasing wet film thickness, the drying time is increased, which leads to a longer reaction time between alkaline slurry and Al-foil. This can be verified from the SEM images in Figure 4, where the thin-film electrodes show fewer pores and cavities in comparison to the thick-film electrode with the same slurry pH value. Since the surface of the deposited slurry was dried faster due to stronger air convection, while the electrode close to the current collector stayed humid for a longer period of time. The formation of hydrogen along the current collector surface continued until the electrode is dried. Thus, the hydrogen bubbles were trapped inside the film during the drying process, forming craters on the surface which could be seen from cross section images in Figure 3b as well as SEM images in Figure 4a,b. The high porosity is disadvantageous for achieving high volumetric energy densities and thus counteracts the benefit of increasing the electrode thickness. Therefore, the slurry pH value adjustment is of great importance for aqueous electrode manufacturing.

Figure 2 shows that slurries with different pH values have similar shear thinning behavior, which is typical rheological behavior of CMC since it is often used as a thickening agent in suspension due to its long-chain molecular and network structures [42]. The long-chain molecules tend to orient themselves in flow direction with increasing shear stress, thus the shear resistance to flow as well as viscosity decreases [43]. It is observed that with an increasing amount of acetic acid addition, the slurry pH value decreases from 12 to 8, while the viscosity increases from 2 to 9 Pa·s at a reference shear rate of 50 s^−1^. The tendency is not the same compared to the viscosity of NMC 111 slurries from Bauer et al. [17], where the viscosity remains constant at around 4 Pa·s in a pH range of 8 to 11. However, the viscosity rise with decreasing pH value from 12 to 8 is consistent with the study on the viscosity of CMC solution under different pH values from Lee at al. [44]. Na-CMC is very sensitive to pH value changes. The variation of pH value affects the protonation of the derivative sites on CMC, which in turn impacts the molecular conformation [45]. In the neutral region (pH value around 7), the dissociation of CMC produces negatively charged polymer chains, the mutual repulsion among carboxylate ions (–COO^−^) results in an expansion of the network chains to form a hydrogel [46], thus leads to high viscosity. When the pH value increases to an alkaline region, sodium ions dissolve from Na-CMC in the water. The number of dissolved sodium ions increases with elevated pH value, resulting in the restraint of the extension of the tangled molecular chain in the hydrogel, therefore, the viscosity decreases [46]. The rheological property of the slurry has a significant influence on the manufacturing process of cathodes, such as mixing and coating. With high viscosity, the sedimentation of active material particles, convection of conductive additives, and binder migration can be limited [47]. Therefore, the addition of acetic acid can not only adjust the slurry pH value to avoid corrosion on the current collector but is also beneficial for thick-film manufacturing.

### 4.2. Electrochemical Performance of Electrodes with Acid Addition

**Figure 9:**
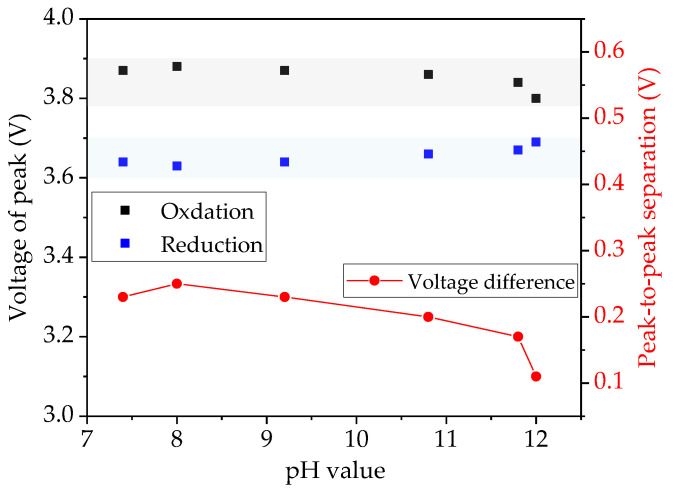
Redox peak voltage extracted from Figure 8 and the redox couple voltage difference of cells with electrodes produced with different slurry pH values.

Figure 8 displays the redox couple of cells containing cathodes produced with different slurry pH values. The peaks measured in cyclic voltammetry (CV) correspond to the oxidation/reduction of Ni^2+^/Ni^4+^ [48]. The voltage of each redox peak in CV measurements and the voltage difference of the redox couple (ΔE_p_) are summarized in Figure 9. With decreasing slurry pH values, the oxidation and reduction peaks shift to higher and lower potential, leading to an increase in voltage difference ΔE_p_ of the redox couple. The rise of ΔE_p_ indicates an increased cell polarization and therefore a sluggish electrochemical reaction [49]. This occurs as a result of the less porous structure in the electrode film. Due to the increased size and number of cavities inside the electrode film with high slurry pH values, the contact surface between electrode and electrolyte is enlarged and the lithium-ions diffusion from the liquid electrolyte to the electrode is enhanced in comparison to electrodes with lower porosity.

**Figure 10:**
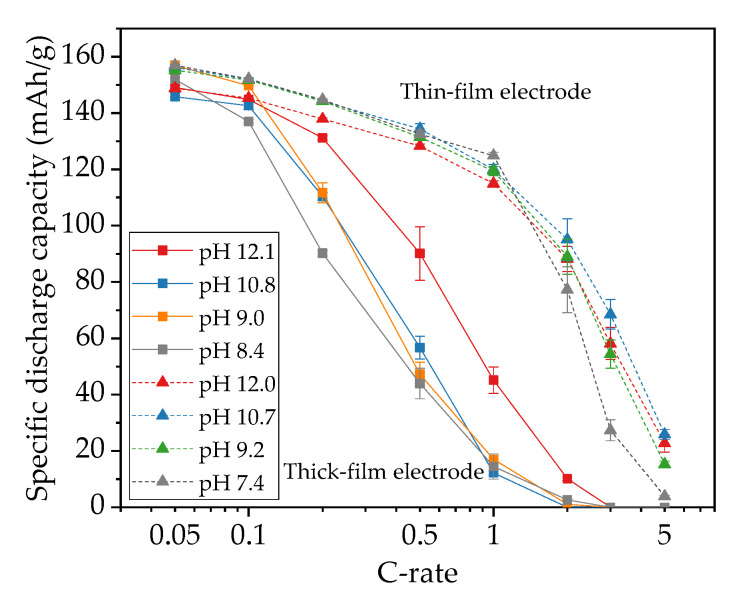
Average discharge capacity as a function of C-rate for cells containing electrodes produced with different slurry pH values.

The functional dependence of discharge capacity vs. C-rate of cells with both thick-film and thin-film electrodes is summarized in Figure 10. A decrease in capacity of about 10 mAh/g for cells with aqueous processed electrodes is observed compared to cells with NMP-based electrodes [30], which may originate from the loss of lithium-ions and dissolution of transition metal ions [21]. In addition, a strong dependence of the specific discharge capacity on electrode thickness and discharge rate was found. At small C-rates of C/20, all cells show a similar initial capacity of about 150 mAh/g. Cells with thick-film electrodes suffer severe capacity fade with increasing discharge current, losing over 60% of the capacity at C/2 compared to capacities at C/20. This may due to the limited lithium-ion charge transfer resistance, local material degradation, and micro-cracks formation on the electrode surface owing to the inhomogeneity of applied current [26]. Although cells with electrodes without acid (slurry pH value of 12.1) show the highest discharge capacity after C/10, the extremely high porosity around 60% hinders the possibility of achieving high volumetric energy densities. Additionally, cathodes with high porosity have reduced adhesion strength to the current collector. The corrosion of the aluminum current collector can lead to a deteriorated cycle stability and a shortened cycle lifetime. Not to mention the shedding of electrode materials during handling, which makes the post-processing of electrodes more difficult, such as calendaring, winding, laser structuring, cutting, and packaging. In addition, the cells containing thick-film electrodes produced with slurry pH values of 8.4 and thin-film electrodes produced with slurry pH values of 7.4 show the lowest discharge capacity above C/10 and C/2, respectively. This indicates that an excessive amount of acid deteriorates the electrochemical performance of cells at high C-rates, leading to increased cell polarization and low capacity retention. However, in the pH range of 4–9, the aluminum current collector is chemically protected due to the existence of a natural alumina passivation layer [39]. Therefore, the pH value of NMC 622 slurry should be adjusted to 9–10 considering the balance between rate capability and current collector corrosion.

**Figure 11:**
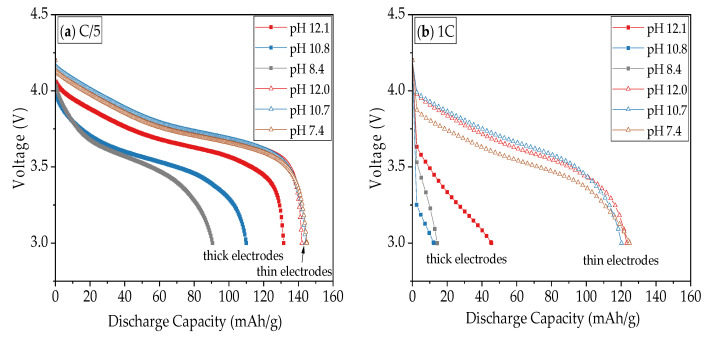

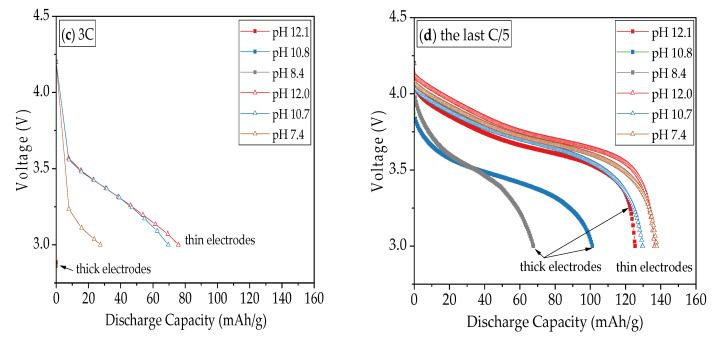
Comparison of the discharge capacity of cells containing thin-film and thick-film cathodes produced with slurry pH values 12, 11, and 8 from Figure 6 at discharge rates of (**a**) C/5, (**b**) 1C, (**c**) 3C, and (**d**) the last C/5.

Figure 11 depicts voltage profiles of cells at selected C-rates of C/5, 1C, 3C, and finally C/5 to discuss in detail the influence of slurry pH values and film thicknesses on the electrochemical performance of the electrodes. At initial C/5, the discharge capacities of thin-film electrodes show no substantial difference, while thick-film electrodes show a 40 mAh/g difference between electrodes with different slurry pH values. With decreasing pH values, the lithium insertion along the voltage plateau is shortened. From 1C to 3C, the discharge capacities of thick-film electrodes drop sharply from less than 50 mAh/g to 0 mAh/g, while the discharge capacities of thin-film electrodes are reduced by 20 mAh/g for each step. The term “IR-drop” refers to the voltage drop resulted from the product of current (I) passing through resistance (R). A sudden voltage drop can be observed at the beginning of the discharge curves in Figure 11. At 1C the IR-drop of cell with thin-film electrode produced with slurry pH value 7.4 is higher (from 4.20 V to 3.87 V) in comparison to cells containing electrodes with slurry pH values 12.0 and 10.7 (from 4.20 V to 3.99 V), which indicates a higher ohmic resistance for the cell containing electrodes produced with lower slurry pH values. After the C-rate is reduced to C/5, thin-film electrodes show 91 ± 4% capacity retention, while thick-film electrodes have a disperse capacity retention of 75%, 92%, and 95% for slurry pH values of 8, 11, and 12, respectively.

The lowering of the discharge curve at a high C-rate is due to cell polarization induced by ohmic resistance, charge transfer resistance, and diffusion overpotential [50]. The electrodes with larger film thicknesses have longer diffusion and migration pathways for lithium-ions to enter and leave the bulk electrode material. The particles in the upper region of the electrode, which are kinetically favored, are intercalating more lithium-ions than the underlying regions. This overpotential in the electrode contributes to additional mass transport losses. The effect is more pronounced for thick-film electrodes, which require higher electrical current density to overcome the mass transport potential. Therefore, the internal resistance of lithium-ion diffusion increases with increasing C-rate. The electronic resistance of the electrode is proportional to its thickness [51], which is in reverse to its electronic conductivity. Thus the thick-film electrode causes an additional ohmic loss. This can be directly observed by the IR-drop at the initial voltage change in each cycle. For example, the voltages for thick-film electrodes decrease from 4.2 V to 3.2–3.6 V at 1C, while the voltages for thin-film electrodes remain at about 4.0 V.

### 4.3. Influence of Laser Structuring on Electrochemical Performance

Laser structuring via ultrafast laser ablation is an appealing method to improve capacity retention at high C-rates, especially for thick-film electrodes. During the formation step at C/20 as shown in Figure 7, the specific discharge capacity of cells containing unstructured electrodes is slightly higher compared to cells with structured electrodes. Such phenomena are also observed in the study from Park et al. using structured NMC 532 electrodes with PVDF as binder material [29]. This might be explained by the formation of a solid electrolyte interface (SEI) between electrode and electrolyte [52]. With a higher slurry pH value, there are more cavities inside the electrode. During cycling, the electrolyte could be pressed into the cavities inside the electrode. The increased interface between the electrode and free electrolyte owing to laser structuring leads to the formation of an extended SEI area. This would consume a higher amount of active material and electrolyte. Another assumption is that the SEI formation might be completed in a short period since the electrolyte wetting of laser structured electrodes is faster, while the SEI in unstructured electrodes could form during the cycling over a longer time period. Electrochemical impedance spectroscopy (EIS) might be applied in order to verify these assumptions.

**Figure 12:**
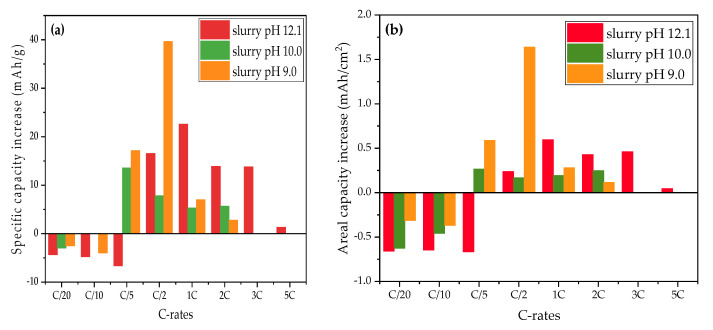
Comparison of (**a**) specific capacity increase and (**b**) areal capacity increase of cells with structured electrodes processed with different slurry pH values compared to cells with unstructured electrodes produced with the same slurry pH value.

Figure 12 shows the increase in specific capacity and areal capacity of cells with structured electrodes compared to cells with unstructured electrodes. The areal capacities can evaluate the capacity increase including the effect of active material loss due to the laser structuring, the areal capacities are calculated from the data shown in Figure 7. When the C-rate increases to C/2, laser structuring begins to show an advantage in capacity with an increase of 14–17 mAh/g (0.2–1.6 mAh/cm^2^) in comparison to cells with unstructured electrodes. This is similar to previous results of cells containing structured electrodes with PVDF as a binder [30]. At C/2, cell containing structured electrodes produced with slurry pH value of 9 retains 40 mAh/g more capacity due to laser structuring, while cells with electrodes produced with slurry pH values of 12.1 and 10 show 16.5 and 8 mAh/g higher capacity in comparison to cells containing unstructured electrodes produced with the same slurry pH value, respectively. This proves that for aqueous processed electrodes with porosities larger than 50% (slurry pH value of 12.1, without acid addition), laser structuring is still feasible to further increase the cell capacity at high C-rates relatively to cells with unstructured electrodes. This may be due to the connection of cavities inside the electrode as shown in Figure 3, which might lead to new diffusion pathways of lithium-ions from electrolyte to the bulk electrode. However, due to the high porosity and the uneven distribution of film thickness, the ablation depth is not stable during laser processing. This might cause local damage to the current collector. Therefore, slurry pH values should be adjusted to 9–10 in order to meet a suitable precondition for subsequent laser structuring.

## 5. Conclusions

Water-based binders CMC and TRD 202A were applied for the preparation of NMC 622 slurry, while acetic acid was added to balance the slurry pH value. Electrodes with different slurry pH values and thicknesses were tape cast on the aluminum foil and calendered afterward. In the last step, laser structuring using an ultrafast laser was performed to improve the electrochemical performance of cells at high C-rates.

The chemical reaction between the slurry without pH modification (pH 12.1) and the aluminum current collector, accompanied by hydrogen release during tape casting, leads to a large number of cavities in the dried cathode film. When the slurry pH is decreased below 10, no cavities inside the thick-film electrode or craters on the electrode surface were observed. With an increasing amount of acid, the viscosity of cathode slurries raised from 2 to 9 Pa·s at a shear rate of 50 s^−1^, which is beneficial for thick-film manufacturing. However, the corrosion and hydrogen generation are stronger with increasing electrode thickness due to the fact that the drying time is necessarily increased with increasing film thickness.

Cyclic voltammetry proves that the addition of acetic acid did not affect the electrochemical reaction of the cathode active material. The voltage difference was increased with decreasing slurry pH values. This occurs as a result of an increased cell polarization caused by a decreased electrode film porosity. A strong impact of the slurry pH value and cathode film thickness on the discharge capacity was observed in rate capability tests. At high C-rates above C/5 for thin-film electrodes and above C/10 for thick-film electrodes, the specific capacity of the electrode with a larger slurry pH value was increased. Thick-film electrodes without acid addition showed the highest capacity, while the electrodes with the highest amount of acid (pH 8.4) retain less capacity at C/10 to 1C. The same tendency is observed for cells with thin-film electrodes. This is due to an improved electrolyte lithium-ion diffusion kinetics inside the electrode with high porosity. Laser structuring is feasible to increase the rate capability of aqueous processed thick-film electrodes, especially at discharge rates above C/2, which is due to an enlarged surface between the active material and free electrolyte, and new diffusion pathways for lithium-ions. In addition, unlike cathodes with high porosity, laser structuring has no significant impact on the post-processing as well as the adhesion of the electrode film to the current collector. Therefore, the pH value of NMC 622 slurry should be adjusted to 9–10, taking into account the balance between rate capability and corrosion of the current collector, as well as the precondition for laser structuring.

## Figures and Tables

**Table 1:** Electrode data after calendering.

Slurry pH Value	Thickness (µm) ^1^	Porosity (%)	Mass Loading (mg/cm^2^)
12.1	240 ± 5	57.5 ± 0.2	36.9 ± 0.2
10.8	189 ± 3	36.6 ± 0.2	43.3 ± 0.1
10.0	200 ± 7	34.6 ± 0.3	47.4 ± 0.2
9.0	189 ± 9	35.1 ± 0.4	44.2 ± 0.3
8.6	186 ± 6	35.5 ± 0.4	43.7 ± 0.3
8.4	191 ± 3	31.7 ± 0.5	47.1 ± 0.4
12.0	130 ± 3	68.1 ± 0.2	13.7 ± 0.1
10.7	82 ± 3	57.7 ± 0.2	10.3 ± 0.2
9.2	51 ± 2	38.8 ± 0.4	11.3 ± 0.1
7.4	76 ± 2	40.7 ± 0.5	12.7 ± 0.2

^1^ The film thickness is the value without the aluminum current collector of 20 µm.

**Table 2:** Active mass loading, areal capacity, and respective mass loss of laser structured electrodes compared to unstructured ones.

Electrode Type	Active Mass Loading (mg/cm^2^)	Areal Capacity (mAh/cm^2^)	Mass Loss (%)
Unstructured, slurry pH 12.1	36.9 ± 0.2	6.13 ± 0.03	−
Structured, slurry pH 12.1	33.3 ± 0.1	5.53 ± 0.02	9.8 ± 1.4
Unstructured, slurry pH 10.0	47.4 ± 0.2	7.88 ± 0.03	−
Structured, slurry pH 10.0	44.0 ± 0.1	7.31 ± 0.02	7.2 ± 0.8
Unstructured, slurry pH 9.0	44.2 ± 0.3	7.35 ± 0.05	−
Structured, slurry pH 9.0	42.7 ± 0.2	7.10 ± 0.03	3.4 ± 0.5

## Data Availability

Data sharing is not applicable to this article.
